# Type I and type IV dermoid sinus with associated cervical spina bifida in a Labrador Retriever mixed breed dog

**DOI:** 10.1002/vms3.1319

**Published:** 2023-11-15

**Authors:** Roman Torres, Carley Giovanella, Kara Sessums

**Affiliations:** ^1^ Department of Neurology and Neurosurgery Mississippi State University College of Veterinary Medicine Mississippi State Mississippi USA; ^2^ Department of Neurology and Neurosurgery Gulf Coast Veterinary Specialists Houston Texas USA

**Keywords:** dermatology, imaging‐CT, imaging‐MRI, neurology, neurosurgery

## Abstract

A 6‐month‐old female Labrador Retriever mix rescued by a local shelter developed respiratory distress and later became tetraplegic. After transferring to a specialty centre, diagnostic imaging (CT and MRI) revealed spina bifida at C3 and dermoid sinuses at the level of C3 and T1. Surgery was performed to remove the dermoid sinuses. The dog was placed on broad‐spectrum antibiotics and a tapering anti‐inflammatory dose of prednisone, postoperatively. Independent ambulation was regained within 14 days with no recurrence of neurologic clinical signs.

## Graphical abstract

Female spayed Labrador Retriever mixed breed dog with a congenital malformation becomes acutely nonambulatory. Advanced diagnostic imaging revealed a cervical dermoid sinus with associated spina bifida at the level C3 and a dermoid sinus at the level of T1.

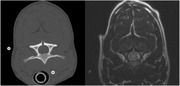

## 1 INTRODUCTION

**FIGURE 1:**
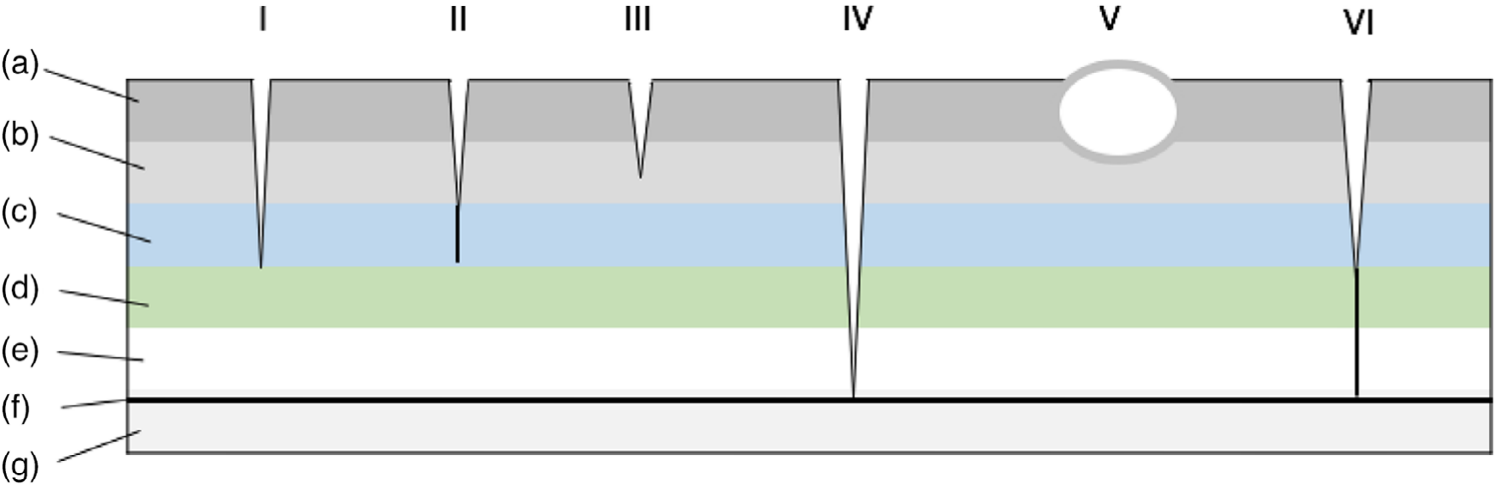
Schematic illustration of the six different types (I–VI) of dermoid sinus (modified from reference Kopke et al.). a (skin), b (subcutaneous tissue), c (musculature), d (supraspinous ligament), e (vertebrae), f (dura) and g (spinal cord).

A dermoid sinus is a hereditary congenital malformation that occurs when the ectoderm fails to separate from the neural tube during early embryogenesis. This leads to the formation of a tubular structure lined with adnexal tissue, including hair follicles, sebaceous tissue and sweat glands that usually extend from dorsal midline to various depths of tissues (Kopke et al., 2019; Kiviranta et al., 2011). There have been six types of dermoid sinuses described (Figure 1A–G). Type I is a tubular sac extending from the epidermis that connects to the supraspinous ligament. Type II is similar to a Type I but more superficial, with a fibrous band at the end of the tubular sac connecting to the supraspinous ligament. Type III extends from the epidermis and terminates superficially to the supraspinous ligament without attaching to it. Type IV extends from the epidermis through the vertebral canal and attaches to the dura. Type V dermoid is a true closed epidermal cyst with no connection to the epidermis. A type VI is a tubular sac that extends from the epidermis and attaches to the supraspinous ligament, continuing as a fibrous band through the vertebral canal and attaching to the dura (Barrios et al., 2014).

There are three subtypes that further classify the dermoid sinus based on location: subtype ‘a’ dorsal midline, subtype ‘b’ head and subtype ‘c’ nose (Barrios et al., 2014; Perazzi et al., 2013). Neurologic signs can occur with Type IV and Type VI dermoid sinuses due to their communication with the subarachnoid space (Barrios et al., 2014; Fleming et al., 2011). Infections of Type IV or Type VI dermoid sinuses may result in meningomyelitis, myelitis, meningitis or abscess formation (Barrios et al., 2014).

Dermoid sinuses have been well documented to occur in dogs with the ridgeback phenotype such as in Rhodesian Ridgeback dogs and their crosses. Dermoid sinuses have been associated with the duplications of fibroblast growth factor genes (FGF3, FGF4, FGF19 and ORAOVI) in these breeds (Fleming et al., 2011; Hillbertz et al., 2007; Kopke et al., 2019). Dermoid sinuses have also been associated with congenital vertebral malformations (hemivertebrae, spina bifida and block vertebrae) in dogs and cats that do not express the ridge phenotype (Barrios et al., 2014; Fleming et al., 2011; Jones et al., 2019; Kopke et al., 2019).

Multiple dermoid sinuses in dogs have been previously reported. One case report describes type Vb and IIIb sinuses affecting the fronto‐occipital region in a Saint Bernard dog, whereas another describes a Chow Chow dog affected with type I, II, III and V dermoid sinuses in the dorsal cervical and craniothoracic regions (Booth, 1998; Perazzi et al., 2013). Complete surgical excision was performed with no returns of clinical signs 5 months following surgery in both cases.

Several reports have documented dogs with a dermoid sinus and associated spina bifida in the craniothoracic region (Kiviranta et al., 2011; Kopke et al., 2019). Rare reports of cats with dermoid sinus and associated spina bifida affecting the cervical region and craniothoracic region have also been documented (Fleming et al., 2011; Kiviranta et al., 2011). This case report describes the history, diagnostic image findings and treatment of a dermoid sinus with associated cervical spina bifida in a Labrador Retriever mix‐breed dog. To the authors knowledge, dermoid sinus with associated spina bifida in the cervical region has not been documented in a dog.

## 2 CASE HISTORY

A 6‐month‐old intact female Labrador Retriever mix weighing 10.4 kg was presented with sudden tetraplegia. She had been rescued as a stray and was initially ataxic upon intake at the shelter two days prior. The dog became lateral recumbent and nonambulatory tetraparetic the morning after intake and developed respiratory distress (full neurologic exam not documented). Treatment included amoxicillin/clavulanate per os (PO) at 18 mg/kg every 12 h for suspected pneumonia and oxygen supplementation. Gabapentin was administered at 9.6 mg/kg PO every 12 h due to the presence of cervical pain. Fenbendazole (unknown dosage) was administered PO to treat a hookworm infection. An electrolyte panel revealed a mild hyponatremia at (136 mmol/L; 145–157), and a mild hypochloremia (103 mmol/L; 105–119). A complete blood cell count (CBC) revealed a mild anaemia with a red blood cell count (4.55 M/μL; 5.65–8.87) and a haematocrit of (29.1%; 37.3–61.7). A neutrophila (23.68 K/μL; 2.95–11.64), a mild monocytosis (3.03 K/μL; 0.15–1.12) and mild eosinopenia (0.01 K/μL; 0.06–1.23) were also noted.

The dog was transferred to a local emergency clinic for continued care. On arrival she was nonambulatory tetraparetic. A repeat CBC revealed the following: red blood cell count (5.25 M/μL; 5.65–8.87), haematocrit (35.2%; 37.3–61.7), neutrophils (22.07 K/μL; 2.95–11.64) and monocytes (4.10 K/μL; 0.16 1.2). A chemistry panel performed revealed the following: amylase (302 U/L; 500–1500) and creatinine (0.3 mg/dL; 0.5–1.8). The dog was started on intravenous (IV) enrofloxacin at 10 mg/kg every 24 h, ampicillin/sulbactam at 30 mg/kg every 8 h, dexamethasone SP at 0.14 mg/kg every 12 h and plasmalyte at 64 mL/h. Radiographs of the neck, thoracic cavity and abdominal cavity were unremarkable. A respiratory disease polymerase chain reaction (PCR) panel returned negative. The dog was transferred to a nearby specialty clinic due to lack of improvement.

At presentation, the dog was alert, responsive, and had a normal cranial nerve exam. However, she exhibited tetraplegia with a positive nociception response in all limbs. The dog could not lift her head and displayed extensor rigidity in the thoracic limbs. Segmental spinal cord reflexes were normal, and there was no pain upon spine palpation. The potential causes for progressive tetraplegia included cervical trauma (vertebral body fracture), congenital malformation, inflammatory processes resulting in a cervical myelitis and/or meningitis secondary to an autoimmune disease (meningoencephalitis of unknown aetiology) or infection (protozoal [*Toxoplasma gondii* or *Neospora caninum*], viral [rabies or less likely canine distemper] or bacterial). Previous cervical radiographs from the emergency clinic did not show vertebral fractures, although the possibility could not be entirely ruled out. The diagnostic plan involved a cervical CT scan to assess bone structures and a cervical MRI to evaluate soft tissue structures.

**FIGURE 2:**
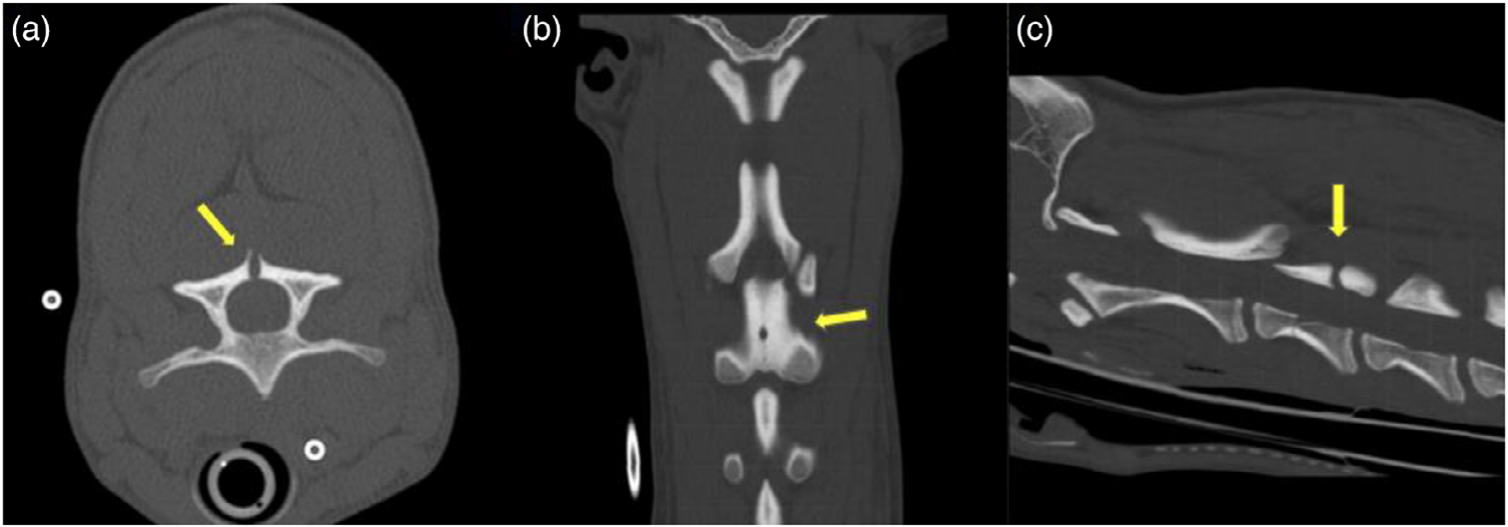
A cervical CT scan of a 6‐month‐old Labrador Retriever mix breed dog. An axial image at the level of C3 (a), a coronal image from C1–C4 (b) and a sagittal image from C1–C4 (c). Incomplete closure of the dorsal aspect of the third cervical vertebrae (yellow arrows) is seen.

**FIGURE 3:**
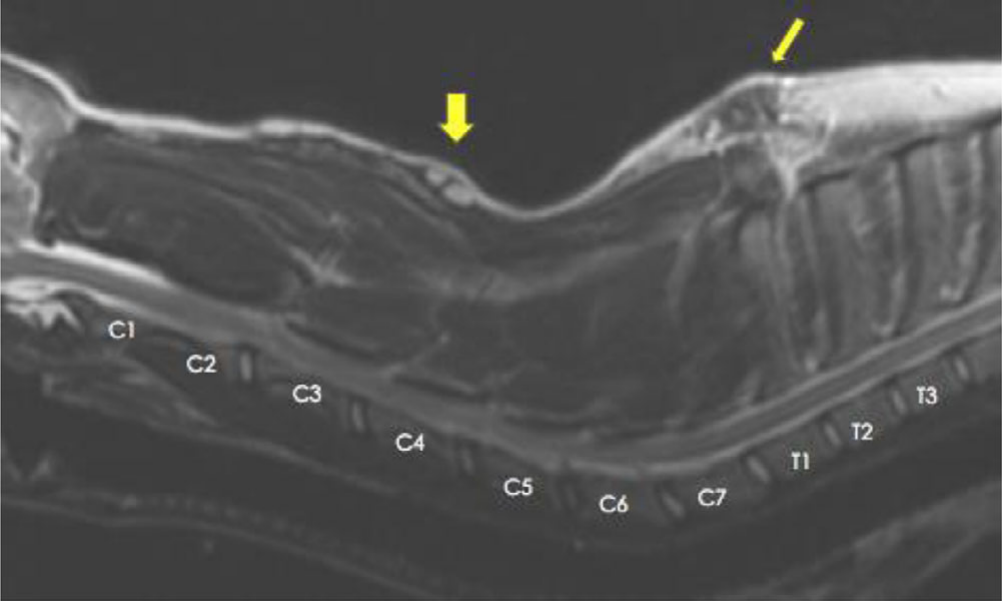
A sagittal T2‐weighted MRI sequence of the cervical and craniodorsal thoracic region from C1–T3. There is an intramedullary hyperintensity of the spinal cord from C2–C6. There is hyperintense lesion in the subcutaneous tissues (short yellow arrow) and a hypointense lesion in the subcutaneous tissue between T1 and T2 (long yellow arrow).

**FIGURE 4:**
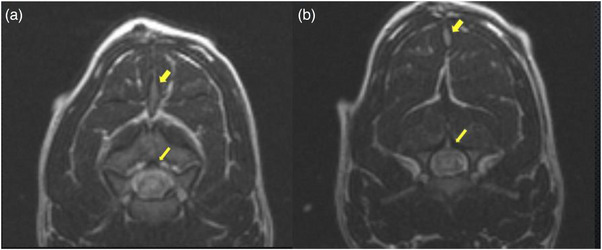
An axial T2‐weighted MRI sequence at the level of C3. The tubular structure within the cervical epaxial muscles at the level of cranial aspect of C3 coursing down towards the spinal canal (short yellow arrow) and expansion of the dorsal subarachnoid space (long yellow arrow) (A). The same tubular structure originating from the subcutaneous tissue at the caudal aspect of C3 (short yellow arrow) and deviation of the dorsal meninges resulting in a triangular shape (long yellow arrow) (B).

**FIGURE 5:**
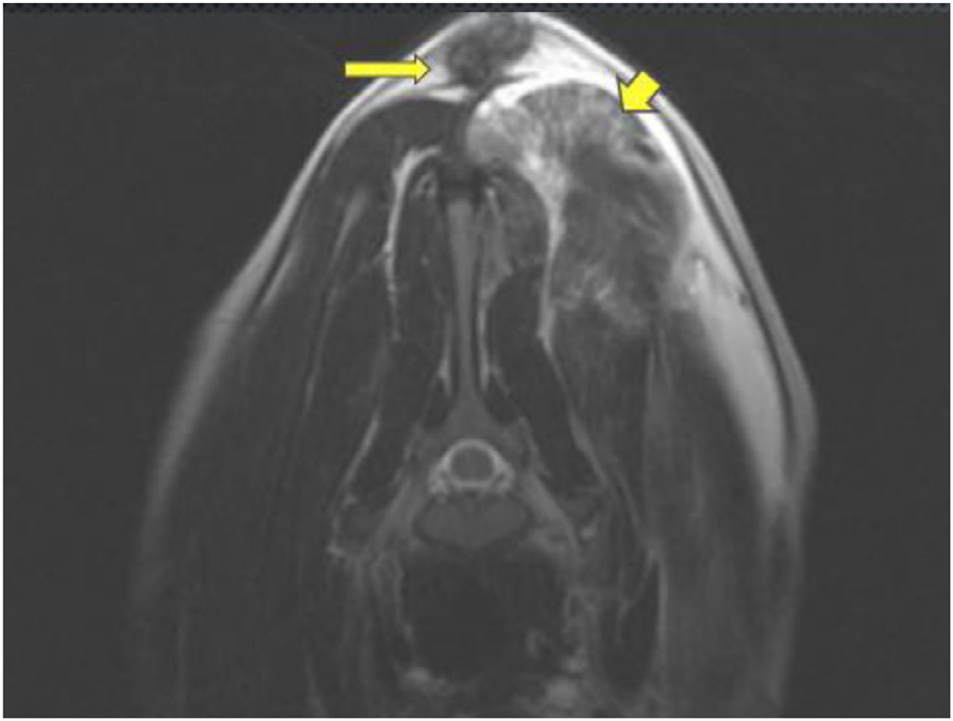
An axial T2 weighted MRI sequence at the level of T1. The tubular structure that originates from the dorsum and extends ventrally to the spinous process of T1 (long yellow arrow). The hyperintense epaxial musculature left to the spinous process of T1 (short yellow arrow).

Results of the cervical CT scan (64 sliced GE Lightspeed Helical) revealed a defect in the dorsal lamina of the third vertebrae consistent with spina bifida (Figure 2A–C). An MRI scan (GE Signa Excite 1.5 T) of the cervical and cranial thoracic region was then performed. A sagittal T2‐weighted sequence revealed an intramedullary hyperintensity from C2 to C6 consistent with spinal cord oedema, a hyperintense lesion within the subcutaneous tissue at the level of C4, and an isointense lesion surrounding subcutaneous tissue between T1 and T2 (Figure 3). An axial T2‐weighted image at the level of C3 revealed a tubular isointense structure originating from a hyperintense lesion in the subcutaneous tissue, which extended deep into the epaxial musculature towards the dorsal aspect of C3 (Figure 4A and B). Additionally, there was a midline defect with dorsal deviation of the meninges at the level of C3 on the same plane that corresponded to the region of spina bifida previously diagnosed via a CT scan, resulting in the subarachnoid space taking up a triangular shape (Figure 4B). An axial T2‐weighted sequence at the level of T1 revealed an isointense structure with an associated draining tract coursing ventrally towards the dorsal part of the spinous process of T1. There was also an associated left sided hyperintensity of the thoracic epaxial musculature at this level that was suggestive of oedema and/or inflammation (Figure 5).

**FIGURE 6:**
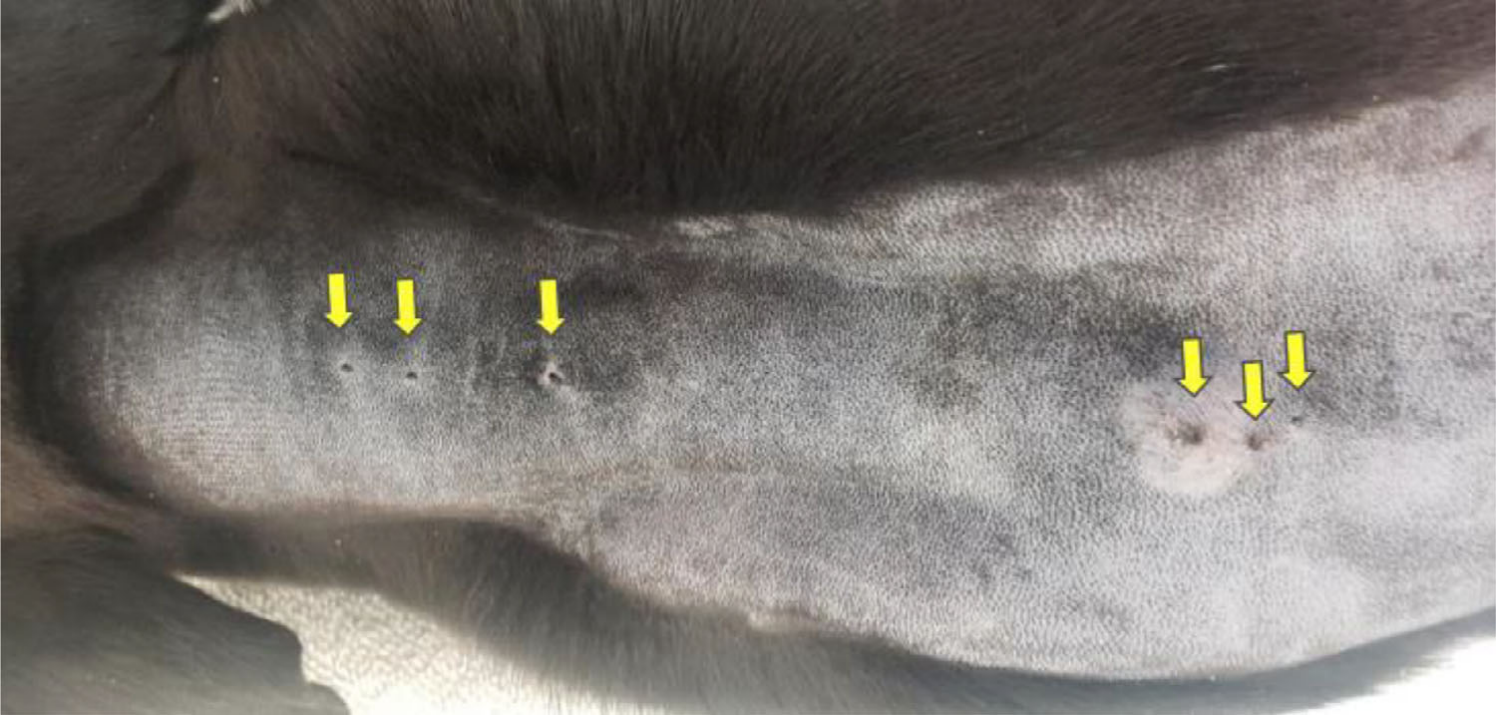
A photograph after the fur from the dorsal cervical to mid thoracic region was removed with clippers. The left side of the image represents the cervical region of the dog. The right side of the image represents the cranial thoracic region of the dog. There are three circular openings in both the cervical and cranial thoracic region (yellow arrows). Palpation of the structures revealed tubular tracts associated with each opening.

A cerebrospinal fluid sample was not collected following advanced imaging. The dog's fur was shaved from the dorsal cranial cervical to mid thoracic region, revealing three circular openings in the cervical area and three circular openings between the shoulder blades. These openings had associated palpable tracts that extended deep into the epaxial musculature and were suspected to be dermoid sinuses (Figure 6). The dog was kept on IV enrofloxacin at 10 mg/kg every 24 h and ampicillin/sulbactam at 30 mg/kg every 8 h over the next 48 h prior to surgery to treat a suspected bacterial myelitis and/or meningitis that resulted from an infected ruptured dermoid sinus. IV Lactated Ringers Solution was administered at a maintenance rate of 60 mL/kg/day. The immunosuppressive dose of dexamethasone SP previously started prior to presentation to the specialty hospital was discontinued and replaced with prednisone at 0.5 mg/kg PO every 12 h. The dog regained motor function in the thoracic limbs and began lifting her head within 24 h. This indicated a need to continue broad‐spectrum antibiotics and a dose reduction of steroids from an immunosuppressive dose to an anti‐inflammatory dose.

**FIGURE 7:**
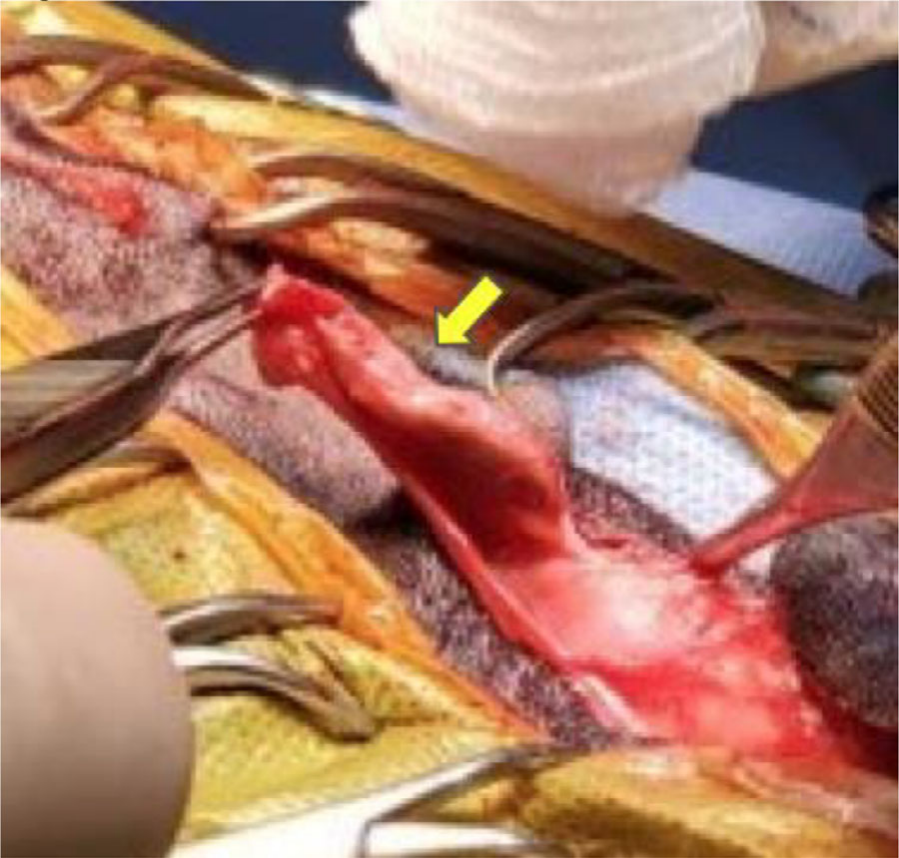
Image of a dermoid sinus in the cervical region being dissected down to the level of C3 (yellow arrow).

**FIGURE 8:**
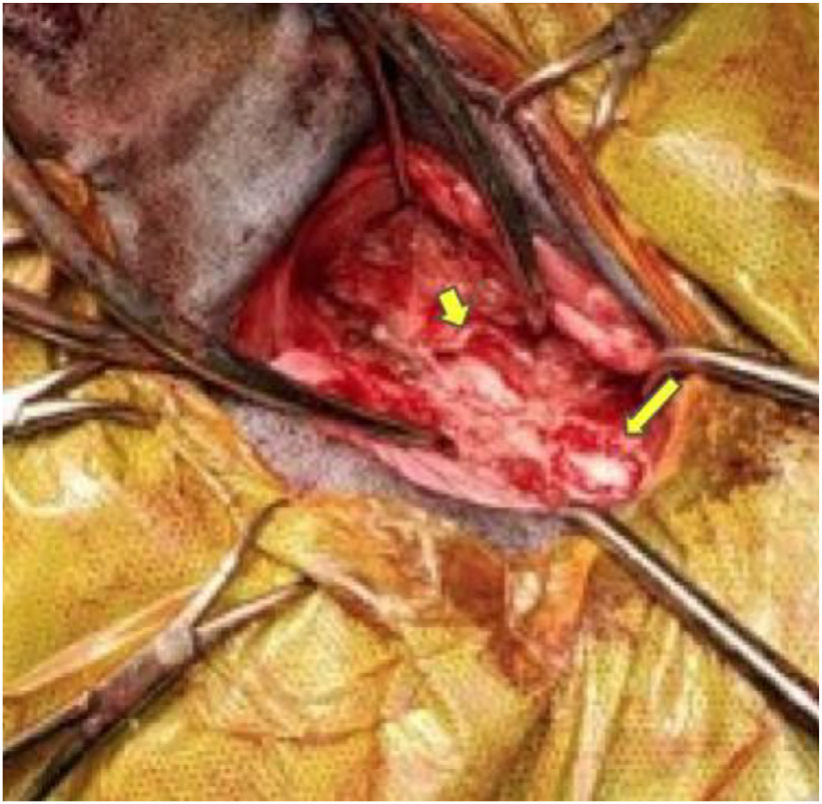
Image after removal of the cervical dermoid sinus and a dorsal laminectomy at C3 (long yellow arrow) and C4 (short yellow arrow).

Surgery was performed to remove the dermoid sinuses via blunt dissection to ensure a complete removal of the abnormal structures (Figure 7). In the cervical region, the dermoid sinus consisted of three small tubular structures merging into a single large tubular structure that extended into the spinal canal through the spina bifida of the C3 vertebrae. This dermoid sinus attached and terminated on the dura of the spinal cord, which was consistent with a Type IV dermoid sinus. A dorsal laminectomy and a durotomy at C3 were performed to remove the cervical dermal sinus attached to the dura, followed by a dorsal laminectomy at C4 due to the presence of purulent material extending from C3 to C4 (Figure 8). Aerobic culture and sensitivity of the purulent material returned negative for bacterial growth 2 days after submission. The dermoid sinus at the level of T1 had three separate small tubular structures that merged into a single larger tubular structure that attached to the spinous process of T1, which was consistent with a Type I dermoid sinus. The proximal edge of the spinous process of T1 was removed along with the dermoid sinus. Tissue samples were submitted for histopathology and were consistent with the diagnosis of a dermoid sinus. The dog was able to right herself into sternal position prior to discharge; she was independently ambulating with no neurologic deficits at her 14‐day post‐operative recheck appointment and was neurologically normal at her 3‐month post‐operative recheck appointment. There was no recurrence of clinical signs 3 months following surgery. Long‐term follow‐up was lost after the dog was adopted out by the local rescue.

## 3 DISCUSSION

A dermoid sinus is a congenital malformation that occurs when the skin ectoderm and the neural tube fail to separate during early embryogenesis (Kopke et al., 2019; Kiviranta et al., 2011). This case report revealed features of a dermoid sinus with associated spina bifida in the cervical region of a mixed breed dog. The diagnosis of a type IV dermoid sinus was made due to the dermoid sinus originating on the surface of the epidermis that continued ventrally through the spina bifida of the third cervical vertebrae, where it attached to the dura of the spinal cord. The dog was also diagnosed with a dermoid sinus type I associated with the spinous process of T1. Previous case reports of dogs with vertebral body malformations concomitant with dermoid sinuses have been described; however, a dermoid sinus with associated spina bifida in the cervical region has not be reported (Barros et al., 2014; Jones et al., 2019; Kiviranta et al., 2011; Kopke et al. 2019).

The dog had a history of cervical hyperesthesia that resolved with gabapentin, dexamethasone SP and gabapentin prior to arriving to the specialty clinic. A C1–C5 myelopathy was suspected due to the dog's intact cranial nerves, normal mentation and tetraplegia with normal segmental spinal cord reflexes. A CT scan performed ruled out a vertebral body fracture; however, it revealed evidence of spina bifida at C3. An MRI was elected as the next diagnostic step to evaluate the cervical and thoracic spinal cord. The MRI highlighted notable observations that reflected inflammation of the spinal cord from C2 to C6, a tubular structure that originated from the dorsal cervical region and extended through the incomplete closure of the lamina of C3 and a tubular structure that originated from the craniodorsal thoracic region and terminated at the level of the spinous process of T1. The tubular structures seen on the MRI were originally suspected of being abscesses with draining tracts sustained from previous wounds. Although there were no notable swelling or fluid filled pockets palpated on the dorsal cervical or cranial thoracic region prior to anaesthesia. The presumptive diagnosis of dermoid sinuses was attained once visualization of symmetrical epidermal orifices in the dorsal neck and cranial thorax, along with palpation of associated tubular stalks occurred. The recommended treatment for a dermoid sinus is complete surgical removal as failure to remove the entire dermoid sinus may result in the reoccurrence of clinical signs. Surgery was elected and the complete removal of the dermoid sinuses, along with a dorsal laminectomy of C3–C4 was performed. Histopathology demonstrated features consistent with a dermoid sinus and secondary moderate, multifocal and lymphohistiocytic inflammation.

It is suspected that the dermoid sinuses developed a local bacterial infection that led to their rupture, resulting in an inflammatory response to the surrounding tissues from the release of keratin and the spread of infection. The dog had a history of respiratory distress while at the animal shelter prior to presentation to the specialty clinic, which improved with time, antibiotics and steroids. A respiratory infectious disease PCR panel and radiographs taken prior to presentation to the specialty hospital did not show evidence of infection or pneumonia, respectively. The respiratory distress was likely a consequence of myelitis and/or meningitis of the cervical spinal cord segment associated with a ruptured and/or infected dermoid sinus in the cervical region.

Injuries to the cervical spinal cord can result in respiratory compromise by disrupting the connection between the bulbospinal neurons in the brainstem and the respiratory motor neurons of the spinal cord (de Lahunta et al., 2021; Jenson et al., 2020). Previous case reports illustrated the diagnosis and characterization of dermoid sinuses using CT scans with contrast; however, IV contrast was not used in this present case and the tubular structures were not visualized on the CT scans alone (Appelgrein et al., 2016; Jones et al., 2019). The diagnosis of dermoid sinuses was made with the MRI images, visualization of openings in the dorsal cervical and craniodorsal thoracic region, and histopathology. Thin stalks of tissue that originated from these openings and diverged deeper into the epaxial musculature increased the suspicion for the diagnosis of dermoid sinuses prior to confirmation via histopathology.

Dermoid sinuses associated with spina bifida and the spinal cord are rare, and based on our search, a dermoid sinus with an associated cervical spina bifida resulting in tetraplegia has never been reported. In this case, surgical management followed by 2 months of post op antibiotic therapy improved the dog's clinical signs without return of neurologic deficits within 3 months.

## AUTHOR CONTRIBUTIONS

*Data curation; methodology; writing – original draft; writing – review and editing*: Roman Torres. *Writing – review and editing*: Carley Giovanella. *Methodology; supervision; writing – review and editing*: Kara Sessums.

## CONFLICT OF INTEREST STATEMENT

The authors declare no conflicts of interest.

## FUNDING INFORMATION

This study was supported by a grant from the Mississippi State Veterinary School.

## ETHICS STATEMENT

The authors confirm that the ethical policies of the journal, as noted on the journal's author guidelines page, have been adhered to. No ethical approval was required as this is a review article with no original research data.

### PEER REVIEW

The peer review history for this article is available at https://publons.com/publon/10.1002/vms3.1319

## Data Availability

Data sharing not applicable to this article as no datasets were generated or analysed during the current study.
